# In1-ghrelin, a splice variant of ghrelin gene, is associated with the evolution and aggressiveness of human neuroendocrine tumors: Evidence from clinical, cellular and molecular parameters

**DOI:** 10.18632/oncotarget.4316

**Published:** 2015-06-18

**Authors:** Raul M. Luque, Miguel Sampedro-Nuñez, Manuel D. Gahete, Ana Ramos-Levi, Alejandro Ibáñez-Costa, Esther Rivero-Cortés, Ana Serrano-Somavilla, Magdalena Adrados, Michael D. Culler, Justo P. Castaño, Mónica Marazuela

**Affiliations:** ^1^ Departamento de Biología Celular, Fisiología e Inmunología, Universidad de Córdoba Hospital Universitario Reina Sofía, Instituto Maimónides de Investigación Biomèdica de Córdoba (IMIBIC), Campus de Excelencia Internacional Agroalimentario (ceiA3), CIBER Fisiopatología de la Obesidad y Nutricón (CIBERObn), Córdoba, España; ^2^ Servicio de Endocrinología y Nutrición, Hospital Universitario de la Princesa, Universidad Autónoma de Madrid, Instituto de Investigación Sanitaria Princesa, Madrid, España; ^3^ Servicio de Patología, Hospital Universitario de la Princesa, Universidad Autónoma de Madrid, Instituto de Investigación Sanitaria Princesa, Madrid, España; ^4^ IPSEN Bioscience, Cambridge, Massachusetts, USA

**Keywords:** ghrelin system, splicing variant, neuroendocrine tumors, aggressiveness, clinical evolution

## Abstract

Ghrelin system comprises a complex family of peptides, receptors (GHSRs), and modifying enzymes [e.g. ghrelin-O-acyl-transferase (GOAT)] that control multiple pathophysiological processes. Aberrant alternative splicing is an emerging cancer hallmark that generates altered proteins with tumorigenic capacity. Indeed, In1-ghrelin and truncated-GHSR1b splicing variants can promote development/progression of certain endocrine-related cancers. Here, we determined the expression levels of key ghrelin system components in neuroendocrine tumor (NETs) and explored their potential functional role. Twenty-six patients with NETs were prospectively/retrospectively studied [72 samples from primary and metastatic tissues (30 normal/42 tumors)] and clinical data were obtained. The role of In1-ghrelin in aggressiveness was studied *in vitro* using NET cell lines (BON-1/QGP-1). In1-ghrelin, GOAT and GHSR1a/1b expression levels were elevated in tumoral compared to normal/adjacent tissues. Moreover, In1-ghrelin, GOAT, and GHSR1b expression levels were positively correlated within tumoral, but not within normal/adjacent samples, and were higher in patients with progressive vs. with stable/cured disease. Finally, In1-ghrelin increased aggressiveness (e.g. proliferation/migration) of NET cells. Altogether, our data strongly suggests a potential implication of ghrelin system in the pathogenesis and/or clinical outcome of NETs, and warrant further studies on their possible value for the future development of molecular biomarkers with diagnostic/prognostic/therapeutic value.

## INTRODUCTION

Neuroendocrine tumors (NETs) comprise a heterogeneous group of relatively rare neoplasias with a primary site originated in the gastrointestinal tract, the pancreas and the lung [1]. Although NETs were initially thought to be uncommon, the incidence and prevalence of these tumors are increasing at a rate of 3–10% per year [2, 3]. Gastro-entero-pancreatic NETs (GEP-NETs) are the most common type of tumors, and have been classified by the World Health Organization into three categories (G1, G2 and G3) based on tumor size, histopathological differentiation, proliferation index (Ki-67), hormonal behavior, neuroendocrine biomarkers (such as serotonin and chromogranin A), direct invasion, and distant metastasis [4–6]. Although this classification is useful to predict prognosis and postoperative recurrence, diagnosis of NETs is frequently delayed several years, probably due to the rare and heterogeneous nature of NETs and the nonspecific initial symptoms. As a result, patients are often diagnosed at advanced stages, when cure is no longer possible, which leads to worsening the patient's quality of life, and to an increase in health care costs [2, 3]. Indeed, despite their identification more than a century ago, NETs remain a poorly understood disease from the clinical and molecular point of view. Accordingly, identification of new molecular diagnostic/prognostic markers, to better define their tumor behavior, from proliferation and metastasis to secretory mechanisms, deem necessary to provide clues for novel therapeutic targets [7, 8].

The ghrelin system is a multifunctional regulatory complex composed of several peptides derived from the ghrelin gene, their known and —expectably— still unknown receptors, and modifying enzymes. This system is widely distributed throughout a variety of tissues, including the gastrointestinal tract (GIT) and the lung, where it can exert a plethora of physiological and pathological actions, including the control of hormonal secretions, energy homeostasis, inflammatory processes, and tumor development and progression [9, 10]. The first peptide identified among the ghrelin gene products was native (canonical) ghrelin, a 28 amino acid (aa) hormone that can be found in acylated (AG; modified with an octanoyl group in the Ser-3) and unacylated forms. AG is the peptide able to bind and activate the only known ghrelin receptor (GHSR1a), which belongs to the family of G-protein-coupled receptors with seven transmembrane domains [9, 11]. The enzyme responsible for ghrelin acylation belongs to the super-family of membrane bound O-acyltransferases (MBOAT) and is termed MBOAT4 or, commonly, ghrelin-O-acyl-transferase (GOAT) [12, 13]. Soon after the discovery of native ghrelin, a number of alternative ghrelin gene-derived peptides and mRNA splice variants were identified [9, 14]. One of these splice forms, the In1-ghrelin variant, is also expressed in the GIT and pancreas [15, 16]. In1-ghrelin peptide shares the initial 13 aa with native ghrelin, including the first 5-aa, which is the minimum sequence required for ghrelin acylation by GOAT and for binding and activation of GHSR1a [9, 15]. However, the aa sequence of the C-terminal tail of In1-ghrelin is totally altered due to the retention of intron 1. Interestingly, not only the ghrelin gene, but also the ghrelin receptor gene can undergo additional processes of alternative splicing to generate the GHSR1b spliced variant which, as the In1-ghrelin variant, also results from retention of an intron generating a truncated G-protein-coupled receptor isoform, with only five transmembrane domains, whose functional activities remain to be fully elucidated [9, 17].

There is growing evidence indicating that changes in the expression of specific components of this system can be associated to the development and/or progression of various cancers. Specifically, several studies have demonstrated that key components of the ghrelin system (e.g. native ghrelin, GHSR1a and GHSR1b) are expressed in various tumors including pituitary adenomas and breast and prostate cancer, thus suggesting a possible autocrine or paracrine role of ghrelin system in the pathogenesis of these tumors (for review see: [9]). In fact, our group recently described that the In1-ghrelin spliced variant could contribute to the pathogenesis of breast tumors and pituitary adenomas [13, 16]. However, there is still limited and conflicting data available on the precise functional roles that play the different components of the ghrelin system in the patho-physiology of cancer development and progression. Moreover, to the best of our knowledge, there is only limited, descriptive evidence on the presence of some components of the ghrelin system in NETs [9, 18, 19], but their exact role, relevance and implication with the development and/or progression of NETs has not been explored in detail.

In this study, we sought to analyze systematically the presence of different components of the ghrelin system (native-ghrelin, GOAT and GHRS1a) and its key splice variants (In1-ghrelin and GHSR1b) in human NETs and, compared their expression with the corresponding adjacent non-tumoral tissues. Additionally, we aimed to evaluate the functional role of In1-ghrelin variant on proliferation, migration, serotonin secretion and expression of proliferative and apoptotic markers in NET cell lines (BON-1 and QGP-1). Results from our study strongly suggest a potential role for the ghrelin system, particularly In1-ghrelin and GHSR1b, in NET pathophysiology.

## RESULTS

A total of 26 patients with GEP-NETs (mean age 58.3 ± 14.4 years old; 57.7% women) were included in the study. Patients' clinical and pathological features are summarized in Table 1. Thirteen patients (50%) had pancreatic tumors (7 non-functional, 5 insulinomas and 1 ectopic Cushing's) and the rest had gastrointestinal carcinoid tumors. Fifteen patients (58%) presented with metastases, the majority of them in regional lymph nodes and/or liver. Pre-surgical chromogranin A was determined in 22 patients, with a mean value of 19.7 ± 21.2 ng/ml [median 15.3 (0–77) ng/ml]. Immunoperoxidase staining for chromogranin A and synaptophysin was positive in all tumor tissues. A Ki-67 immunoreactivity level >2% was observed in 7 out of the 14 available samples [mean Ki-67 index 10.7 ± 23.3%; median 2.5 (2–90) %].

**Table 1:** General characteristics of the patient population and samples

Patient Baseline characteristic (*n* = 26)	
**Gender**	
Male	11 (42.3%)
Female	15 (57.7%)
**Age, years**	
< 55	11 (42,3%)
≥ 55	15 (57,7%)
**Stage (ENETS)**	
I	6 (26.1%)
II	5 (19,2%)
III	2 (7.7%)
IV	13 (50%)
**Sample characteristics (*n* = 72)**	
**Sample type**	
Primary tumor tissue	26 (36.1%)
Metastatic tissue	16 (22,2%)
Non-tumor adjacent tissues	30 (41,7%)
**Primary site**	
Pancreatic neuroendocrine tumor	13 (50%)
Carcinoid gastrointestinal NET	13 (50%)
**Primary tumor size, cm**	
< 3.0	16 (62.5%)
≥ 3,0	10 (38.5%)
**Grading (WHO 2010 criteria)**	
Low	7 (26.9%)
Intermediate	6 (26.1%)
High	1 (3.8%)
Unknown	12 (46.2%)

Abbreviations: NET = Neuroendocrine tumor; WHO = World Health Organization; GOAT = Ghrelin O-acyltransferase.

### Ghrelin system components are overexpressed in GEP-NETs in comparison to normal tissues

qPCR analysis performed in GEP-NET tissue samples revealed that native ghrelin was expressed in 35.7% (*n* = 15/42), its cognate GHSR1a receptor in 88.1% (*n* = 37/42) and GOAT in 85.7% (*n* = 36/42) (Table 2). Interestingly, we found a high expression of the splicing variants of the ghrelin system in GEP-NETs. Specifically, the In1-ghrelin variant was expressed in more GEP-NET samples than native ghrelin [81% (*n* = 34/42) vs. 35.7% (*n* = 15/42); mean mRNA copy number: 0.038 ± 0.007 vs. 0.00283 ± 0.00025, respectively; 13-fold higher, (*p* < 0.001; Table 2). Similarly, although truncated GHSR1b variant was expressed in a similar percentage of GEP-NET tissues as GHSR1a [92.9% (*n* = 39/42) vs. 88.1% (*n* = 37/42)], the expression levels of this spliced receptor variant were significantly higher than the expression of GHSR1a in GEP-NETs (mean mRNA copy number: 1.764 ± 0.400 vs. 0.215 ± 0.098, respectively; 8-fold higher, (*p* < 0.001; Table 2).

**Table 2:** Percentage (%) of control normal-adjacent tissues and NET tissues expressing the components of the ghrelin system and mean mRNA copy number of each component ± SEM (adjusted by β-actin expression)

	*GOAT*	*Ghrelin*	*In1-ghrelin*	*GHSR1a*	*GHSR1b*
Control	22/30 (73.3%)	6/30 (20.0%)	15/30 (50.0%)	8/25 (32.0%)	21/30 (70.0%)
	0.259 ± 0.077	0.003 ± 0.002	0.008 ± 0.003	0.093 ± 0.063	0.409 ± 0.111
Tumor	36/42 (85.7%)	15/42 (35.7%)	34/42 (80.9%)	37/42 (88.1%)	39/42 (92.9%)
	0.3499 ± 0.055	0.0028 ± 0.002	0.0384 ± 0.007	0.2158 ± 0.097	1.764 ± 0.400

**Figure 1:**
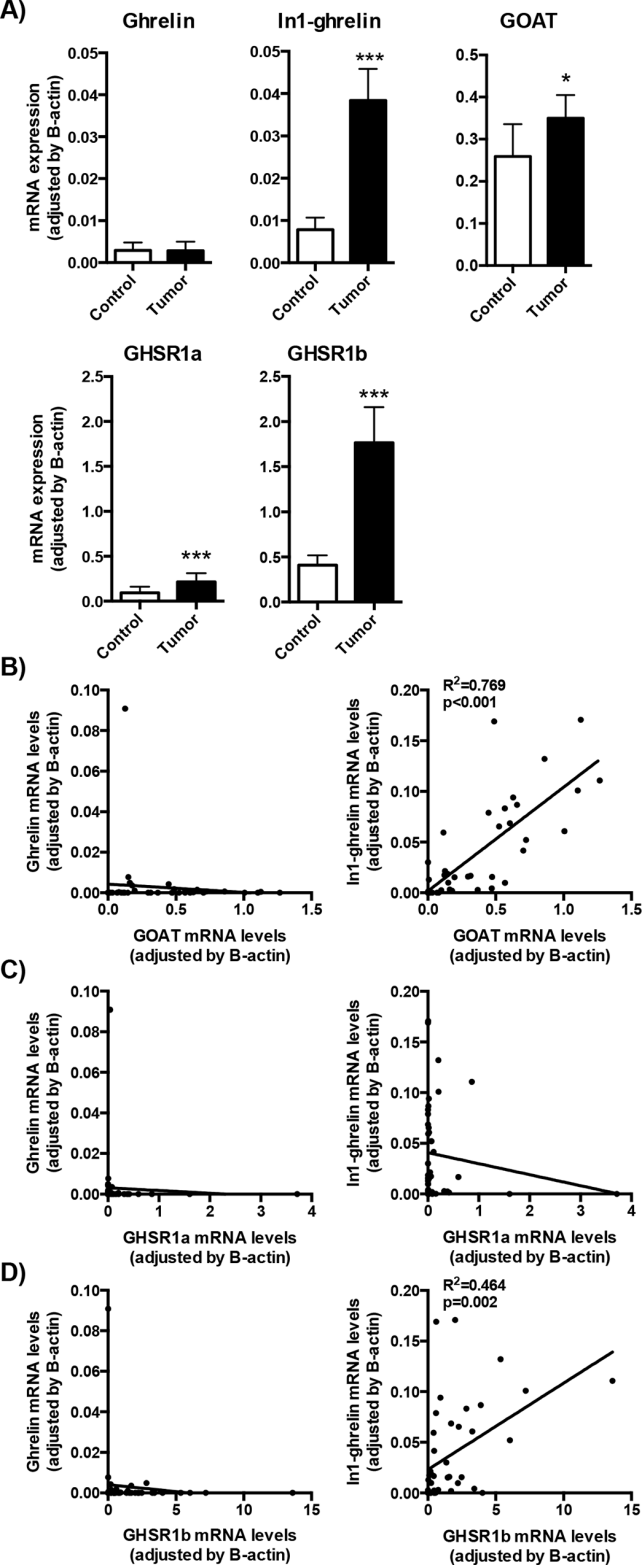
Expression of ghrelin system components in NETs **A.** The mRNA expression levels of the ghrelin system components (ghrelin, In1-ghrelin, GOAT, GHSR1a and GHSR1b) were determined by qPCR in a battery of NETs and compared to their adjacent non-tumoral control tissues. **B.** Correlation between ghrelin or In1-ghrelin levels and GOAT expression levels in NET samples. Correlation between ghrelin or In1-ghrelin levels and GHSR1a **C.** or GHSR1b **D.** expression levels in NET samples. Data represent mean ± SEM. Asterisks (*p < 0.05; ***p < 0.001) indicate values that significantly differ from the adjacent non-tumoral control tissues.

We observed a striking dysregulation of the expression patterns of several components of the ghrelin system in GEP-NETs in comparison to non-tumor adjacent tissues. Specifically, whereas mean mRNA levels of native ghrelin were not different between tumoral and non-tumoral adjacent samples (Figure 1A), we found that mean expression levels of GHSR1a, GOAT, and also of the splice variants In1-ghrelin and GHSR1b, were significantly increased in GEP-NET tissues compared to the non-tumoral adjacent regions (Figure 1A). Interestingly, mRNA expression levels of In1-ghrelin strongly correlated with those of GOAT in GEP-NETs (Figure 1B). This finding was not observed, however, for native ghrelin. In addition, whereas GHSR1a mRNA levels did not correlate with native ghrelin or In1-ghrelin levels, we found that expression levels of GHSR1b paralleled those of In1-ghrelin. However, once again, this was not observed for native ghrelin (Figure 1C). Furthermore, GHSR1b and GOAT expression levels were positively correlated in tumors (Figure 1D).

Further sub-analyses of the expression of the ghrelin components according to tumor site (primary or metastatic tissues) showed a consistent increase of In1-ghrelin as well as of the two ghrelin receptors (GHSR1a and GHSR1b) in both categories, while GOAT and native ghrelin expression levels were elevated only in metastatic tissues (Supplemental Figure 1).

### In1-ghrelin, GOAT and GHSR1b expression is associated to worse patient outcome

**Figure 2:**
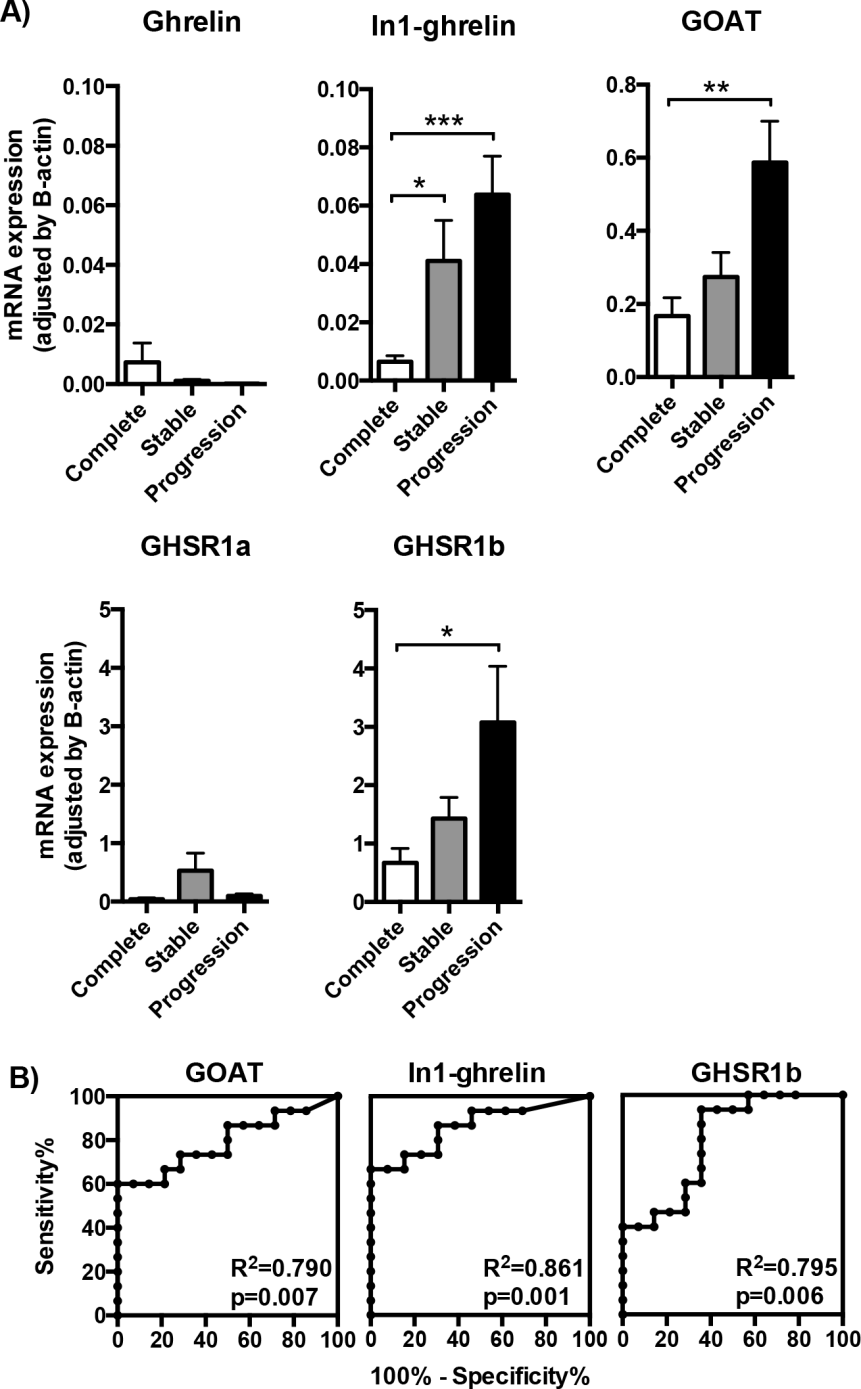
Expression of ghrelin system components in the tumoral samples of patients with different outcome **A.** The mRNA expression levels of the ghrelin system components (ghrelin, In1-ghrelin, GOAT, GHSR1a and GHSR1b) determined by qPCR in the tumoral samples were classified depending on the outcome of the patient (complete remission, stable disease or progression of the tumor). **B.** Receiver operating characteristic (ROC) curve analysis to determine the accuracy of ghrelin system components (GOAT, In1-ghrelin and GHSR1b) as diagnostic test to discriminate between NETs with complete remission or progressive disease [the closer the ROC curve is to the upper left corner of the graphic (i.e., higher sensitivity and specificity), the higher the overall accuracy of the marker used]. Data represent mean ± SEM. Asterisks (*p < 0.05; **p < 0.01; ***p < 0.001) indicate values that significantly differ from the NETs with complete remission.

Patients were grouped into three categories according to their clinical outcome (complete tumor remission, stable disease or progressive disease), and the relevance of the increased expression levels observed for some of the components of the ghrelin system was evaluated accordingly. No significant differences were found in mean expression levels of native ghrelin and GHSR1a between the three groups (Figure 2A). In contrast, higher mRNA levels of GOAT, In1-ghrelin and GHSR1b were associated to a worse clinical outcome (Kruskall-Wallis one-way ANOVA test: *p* = 0.014, *p* = 0.004 and *p* = 0.014, respectively; Figure 2A). These differences were particularly noticeable when comparing tumor samples from patients with complete remission and those with progressive disease (by Dunn's multiple comparisons test: *p* < 0.05, *p* < 0.01 and *p* < 0.05; or by Student's *t*-test: *p* < 0.01, *p* < 0.001 and *p* < 0.05, respectively; Figure 2A). In fact, ROC analysis of all the components of the ghrelin system showed that expression of GOAT, GHSR1b, and, specially, In1-ghrelin, discriminated between categories of clinical outcome (Figure 2B). Conversely, native ghrelin and GHSR1a expression showed a poor ability to distinguish between the two diagnostic groups (ROC curves similar to the reference line; data not shown). Importantly, as illustrated in Figures 2A and 2B, expression of the In1-ghrelin variant was the best and most accurate marker to distinguish between patients presenting complete disease remission or progressive disease. Furthermore, In1-ghrelin expression levels, but not those of GOAT or GHSR1b, were also significantly higher in tumor samples from patients with stable disease, compared to those with complete disease remission (*p* < 0.05 by Student's *t*-test; Figure 2A). Taken together, these findings invite to hypothesize that In1-ghrelin could serve as a potential new molecular diagnostic/prognostic marker and/or a tool to identify new therapeutic targets for the treatment of GEP-NETs and, hence, should be explored further.

### Expression of the splice variants of the ghrelin system, especially In1-ghrelin, is associated with features of malignancy in patients with GEP-NETs

**Figure 3:**
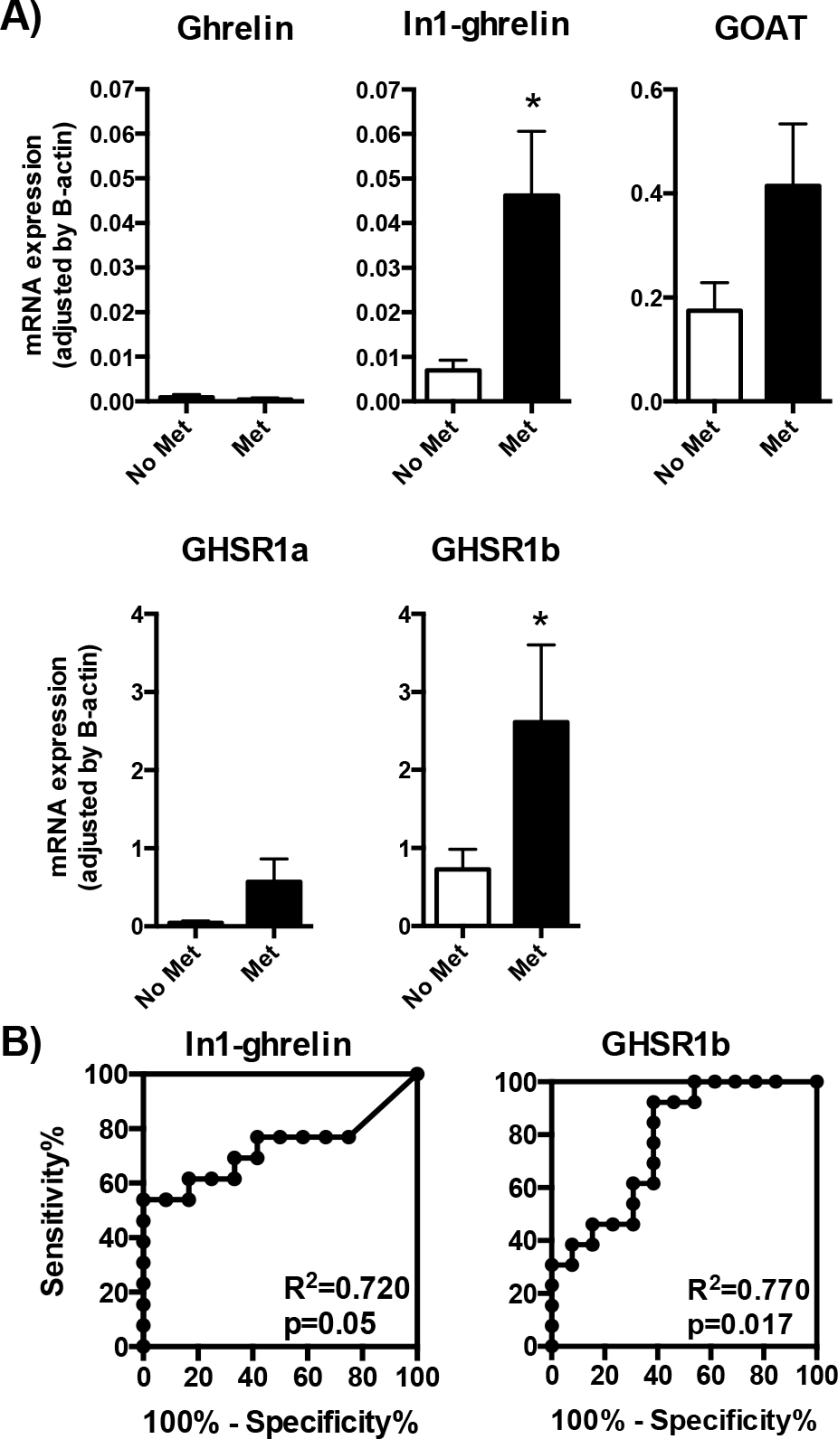
Expression of ghrelin system components in primary NETs with or without metastasis **A.** The mRNA expression levels of the ghrelin system components (ghrelin, In1-ghrelin, GOAT, GHSR1a and GHSR1b) determined by qPCR in the tumoral samples were classified depending on the presence or absence of metastasis. **B.** Receiver operating characteristic (ROC) curve analysis to determine the accuracy of ghrelin system components (In1-ghrelin and GHSR1b) as diagnostic test to discriminate between the metastatic capacities of the NETs. Data represent mean ± SEM. Asterisks (*p < 0.05) indicate values that significantly differ from the NETs without metastasis.

Clinical outcome of patients with GEP-NETs is believed to depend on the aggressiveness of the tumor itself. Therefore, we explored the putative associations between the expression levels of the ghrelin system components in GEP-NET tumor samples and the corresponding clinical, anatomical and pathological characteristics of patients. In this regard, we evaluated expression levels in primary tumors of patients who developed metastases, compared to those who did not. Interestingly, we found that only the expression levels of the spliced variants of the ghrelin system, In1-ghrelin and GHSR1b, were significantly elevated in the primary tumors of patients that developed metastasis, as compared to those that did not (Figure 3A). In fact, although similar, but not statistically significant, trends were observed for GHSR1a and GOAT expression (Figure 3A), ROC analysis of all these components confirmed that only the expression of In1-ghrelin and GHSR1b splicing variants could discriminate between these two diagnostic groups (development of metastasis vs. no metastases; Figure 3B).

Because expression of the In1-ghrelin variant was consistently elevated in all GEP-NET types (primary and metastasis tissues), and that it was associated to expression levels of GOAT and GHSR1b in GEP-NET tissues, we deemed of great interest to explore the putative role of this novel In1-ghrelin variant in the pathophysiology of these tumors, especially given the fact that this was not observed for native ghrelin.

### Overexpression of In1-ghrelin is associated with increasing features of aggressiveness in human NET cells

**Figure 4:**
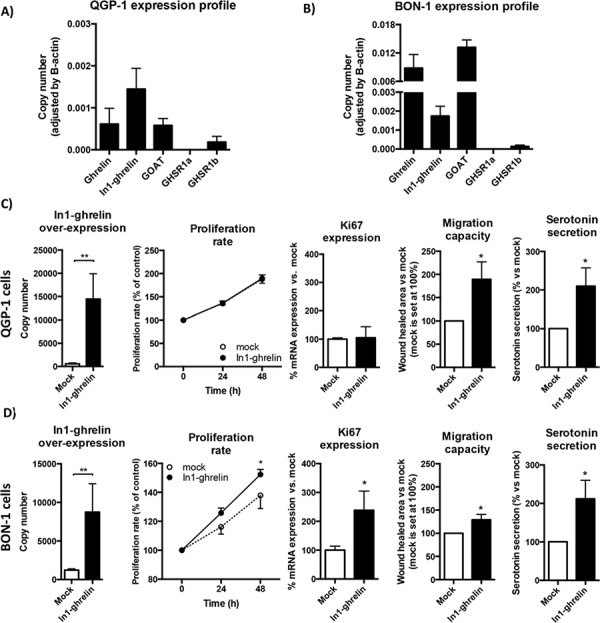
Effects of In1-ghrelin overexpression in NET-derived cell lines **A.** Normalized levels of ghrelin system components (ghrelin, In1-ghrelin, GOAT, GHSR1a and GHSR1b) in QGP-1 NET cell lines and **B.** BON-1 NET cell lines. Functional assays in In1-ghrelin transfected QGP-1 cell lines **C.** and BON-1 cell lines **D.** compared to mock control cells. Panel order from left to right: In1-ghrelin overexpression; proliferation rate; Ki67 expression; migration capacity; and serotonin secretion. Data represent mean ± SEM of *n* = 3 − 6 independent experiments. Asterisks (*p < 0.05; **p < 0.01) indicate values that significantly differ from mock control cells.

To examine the possible functional effects of the In1-ghrelin variant on NET cells malignancy features, we used two commonly accepted models for NET cell studies: BON-1 and QGP-1 cell lines (Figure 4). qPCR analysis performed in both cell lines revealed that the majority of the components of the ghrelin system were expressed, at different levels, except for GHSR1a (i.e. its expression was under the detection limit) (Figure 4A and 4B). In particular, In1-ghrelin was the ghrelin system component most abundantly expressed in QGP-1 cells, followed by native ghrelin and GOAT, and by a modestly expressed GHSR1b (Figure 4A). Conversely, native ghrelin and GOAT were the most predominantly expressed components of the ghrelin system in BON-1 cells, followed by In1-ghrelin expression, and by a much lower GHSR1b expression level (Figure 4B).

We then overexpressed In1-ghrelin in both cell lines by transfection with a specific expression vector containing the appropriate coding region of the In1-ghrelin variant. We verified an increased number of mRNA copies of In1-ghrelin compared to mock-transfected controls by qPCR (Figures 4C and 4D: first left panels). This strategy revealed that In1-ghrelin overexpression increased the proliferation rate of BON-1, but not of QGP-1 cells, compared to their corresponding mock-transfected control cells (Figures 4D and 4C, respectively: second panels). In agreement with this finding, a significant increase in the expression of the proliferation marker Ki67 was observed in BON-1, but not in QGP-1, cells transfected with In1-ghrelin compared with mock-transfected cells (Figures 4D and 4C, respectively: third panels). Additionally, we also investigated whether In1-ghrelin overexpression exerted apoptotic effects by measuring the expression levels of two apoptosis-related markers, TP53 and Bcl2, in both cell lines. Our data indicated that overexpression of In1-ghrelin did not alter the expression levels of these two apoptosis markers in NET cell lines (Supplemental Figure 2), suggesting that In1-ghrelin is not involved in the control of the expression of these apoptosis-related marker.

Interestingly, further functional assays revealed that overexpression of In1-ghrelin significantly stimulated the migration capacity of both QGP-1 and BON-1 cells, as assessed by wound healing technique (Figures 4C and 4D, respectively: fourth panels). Furthermore, In1-ghrelin overexpression also increased significantly serotonin secretion in both cell lines as compared to their corresponding mock-transfected control cells (Figures 4C and 4D: fifth panels).

## DISCUSSION

NETs have been commonly considered a rare and heterogeneous group of neoplasms [1]. However, their prevalence is higher than previously thought according to recent data from the US Surveillance Epidemiology and End Results [2]. Although in recent years there has been significant progress in NET medical treatment, with the emergence of multiple active therapies [20], these strategies are still limited and unsatisfactory. Therefore, it is crucial to identify new molecular biomarkers for these tumors, which would help to refine their diagnosis, to better predict their prognosis and tumor behavior, and provide tools to develop novel therapeutic targets.

Earlier studies suggested that the ghrelin system, which participates in various endocrine-related tumors [9, 10, 21], might also play a relevant role in NET pathophysiology. Indeed, some of its components have been previously detected in normal and tumoral tissues of the GIT [9, 22]. Specifically, ghrelin and GHSR1a are expressed in gastric [18, 23–25], intestinal [23, 26], pancreatic [18, 25, 27] and/or lung [28] NETs, although the expression levels and the percentage of tumors expressing native-ghrelin or GHSR1a varied substantially depending on the tissue analyzed, and on the detection method used. Moreover, to date, it is still unclear whether the expression profiles of these or other components of the ghrelin system are altered in NETs. Actually, to the best of our knowledge, the present report provides the first systematic analysis of the expression of different components of the ghrelin system in human NET tissues as compared to their corresponding adjacent non-tumoral tissues, and in relation to clinical parameters of the patients.

Our results revealed that the expression profile of ghrelin system is markedly altered in NETs compared with control adjacent tissues, and that this is dependent on the specific component examined, and on the tumor site analyzed (primary vs. metastatic tissues). Specifically, In1-ghrelin, GHSR1a/1b and GOAT, but not of native-ghrelin, expression was increased in NETs compared with control adjacent tissues, both in primary and in metastatic sites. Moreover, expression was higher in metastatic tissue when compared to the primary site, suggesting that these components might exert a functional role in the NETs pathology. Interestingly, results differed in the case of GOAT, which was only overexpressed in metastatic tissues. Our results further indicate that In1-ghrelin is the variant predominantly expressed in NET tissues, in terms of number of NET samples and expression levels (i.e. 13-fold higher than those of native-ghrelin). This observation compares favorably with previous reports indicating that In1-ghrelin, but not native-ghrelin, expression is consistently increased in other endocrine-related tumors compared with normal tissues, including human breast [15] and pituitary [21] tumors. Hence, it seems reasonable to suggest that overexpression of this In1-ghrelin might be a common cellular/molecular signature across various endocrine-related tumors.

This study also demonstrates that a high proportion of NETs express GOAT, an enzyme that acylates native-ghrelin, and presumably also In1-ghrelin variant, which is an essential step for ghrelin to bind GHSR1a and to exert thereby its neuroendocrine activities [12, 13, 21]. The remarkable expression of GOAT in NET tissues compared with that in adjacent-control tissues, is in accordance with recent findings in breast cancer tissues where GOAT mRNA was highly expressed compared with control tissues [15], like in prostate cancer cell lines compared with normal prostate epithelial-derived cell lines [29]. Expression of GOAT enzyme, as well as of native-ghrelin and In1-ghrelin in NETs, supports the notion that the ghrelin axis might exert active autocrine/paracrine roles in these tissues. Actually, expression levels of GOAT in NETs positively correlated with In1-ghrelin, but not with native ghrelin expression, suggesting that In1-ghrelin might be the main ghrelin gene variant functionally linked to GOAT in these tumors, and reinforcing the idea that an autocrine/paracrine circuit involving these two components may operate in NETs, especially in metastatic tissues, where both components were highly expressed compared with adjacent-control tissues.

Regarding the expression of receptors, our analyses revealed that NETs not only expressed the canonical, full length GHSR1a, but also GHSR1b, which is in line with previous reports showing that the latter is present in intestinal carcinoids [23] or pancreatic NETs [25]. Moreover, our study is the first to demonstrate that expression levels of both receptors are significantly elevated in NET tissues compared with non-tumoral adjacent tissues. Specifically, the splice variant GHRS1b was the dominant ghrelin receptor isoform expressed in NET tissues, as its mRNA levels were markedly higher (8-fold) than those of GHRS1a. This data is reminiscent of earlier reports showing that GHSR1b seems to be selectively expressed in prostate hyperplasia [30], as well as in breast carcinomas [15], where its abundance shows a strong correlation with In1-ghrelin expression levels. Interestingly, although GHSR1b expression levels in NETs were significantly higher than those of GHSR1a, both receptors were expressed in a similar percentage of tumor tissues, where their expression levels tended to correlate (*p* = 0.06), suggesting a potential functional association between both receptors in NETs. Although the actual (patho)physiological role of GHSR1b is still unknown, it has been reported that GHSR1b can heterodimerize with GHSR1a, promoting translocation of the receptor complexes, and acting thereby as a dominant-negative receptor for the canonical signaling functions of GHSR1a [31, 32]. In this scenario, it is tempting to speculate that the striking change in the mRNA pattern of GHSR1a and GHSR1b found in NET tissues (i.e. significant high level of GHSR1b relative to GHSR1a) may translate into a relevant functional role in this pathology (e.g. by disrupting the normal ghrelin/GHSR1a signaling). In fact, whereas GHSR1a mRNA levels do not correlate with those of native ghrelin or In1-ghrelin in NETs, GHSR1b expression levels did parallel those of In1-ghrelin (*p* = 0.002), but not those of native ghrelin. This suggests that In1-ghrelin could be a relevant ghrelin gene-derived variant functionally linked to other components of the ghrelin system [i.e. with GOAT (as discussed previously) and GHSR1b] in NETs and, that the cellular machinery responsible for the intron retention processes is altered [33].

Further support to the notion that the splice variants In1-ghrelin and GHSR1b may promote NETs pathogenesis derives from three lines of evidence. First, from the observation that there is not only a quantitative predominance of the expression of these splice variants over their respective canonical counterparts in NET tissues, but also that the ratios between In1-ghrelin/native-ghrelin, and between GHSR1b/GHSR1a are augmented in NET tissues as compared with that found in adjacent-control tissues (∼5-fold and 2-fold higher for In1-ghrelin/native-ghrelin and GHSR1b/GHSR1a ratio, respectively). The second line of support is provided by the direct association of In1-ghrelin, GHSR1b and GOAT expression levels —but not of native-ghrelin and GHSR1a— with a worse clinical outcome, i.e. tumors of patients with progressive disease have higher levels of these components vs. patients with complete remission of the tumor. These results are also consistent with previous reports showing that native-ghrelin expression in pancreatic NETs is not clinically associated with tumor size, grade or stage [32], and with studies showing that plasma native-ghrelin concentrations lie within the normal range in patients with NETs [34, 40, 41] (native-ghrelin elevation has only been reported in three cases of ghrelinomas [41–43]). Additionally, we found that only the expression levels of In1-ghrelin and GHSR1b were significantly elevated in the primary tumors of patients that developed metastasis, as compared to those that did not. And thirdly, ROC curve analysis indicated that only the expression of In1-ghrelin and GHSR1b could discriminate between the different diagnostic groups according to follow-up outcome (tumor remission, stable disease, or progressive disease) and malignancy features (expression in primary tumors of patients that developed metastasis compared to those that did not develop metastasis), whereas GOAT expression could only discriminate between the diagnostic groups of clinical outcome of patients. These findings suggest a putative utility of these splicing variants as tools to identify novel biomarkers to refine diagnosis and predict prognosis of NETs, and for the development of new therapeutic tools for the management of human NETs.

Based on previous results showing that In1-ghrelin variant can promote features of aggressiveness in breast cancer [13] and pituitary tumor cells [16], and on the results of our study, which demonstrated that the expression of the In1-ghrelin variant is a valuable marker to classify patients according to prognosis, we further explored the functional role of In1-ghrelin in two NET cell models. *In vitro* data revealed that overexpression of In1-ghrelin enhanced features of aggressiveness in human NET cells (i.e. increased proliferation rate and expression of the Ki67, as well as migration capacity and serotonin secretion) which demonstrates that this splice variant is functionally active in NET cells. These results support and extend previous data from our group showing that In1-ghrelin influences key, clinically relevant processes, such as proliferation or hormone secretion in other endocrine-related pathologies as breast cancer [15] and pituitary adenomas [21], further suggesting that overexpression of In1-ghrelin might be a common cellular/molecular signature across different endocrine-related tumors that is directly associated to the aggressive features of these pathologies.

Altogether, our results indicate that the ghrelin system, specially the splicing variants (In1-ghrelin and GHSR1b), is dysregulated in human NETs, where they may exert a relevant pathophysiological role. Specifically, the observations indicating that In1-ghrelin expression is correlated with a worse patient's outcome, increased malignancy, and features of aggressiveness in human NET cells support the idea that this system could potentiate the pathogenesis of NETs, and may provide useful tools to identify new diagnostic/prognostic biomarkers and explore novel therapeutic molecular targets in human NETs.

## MATERIALS AND METHODS

### Study population and samples

We collected data from 26 patients with GEP-NET who underwent surgery at our Hospital from 2001 to 2009 [mean age 58.4 ± 14.4 years, 15 females (57.7%)]. All patients were carefully screened for the presence of other malignancies, and special attention was paid to exclude an association with neurofibromatosis, multiple endocrine neoplasia type 1, or Von Hippel-Lindau syndrome. Only one patient was carrier of a MEN-1 gene mutation.

We used clinical records to collect full medical history of all patients. Subjects were classified following ENETS and WHO criteria (tumor site and size, angioinvasion, infiltration level, cell proliferation index, immunohistochemical phenotype, and metastases) [34, 35]. In addition, GEP-NETs were classified according to histopathology features as well-differentiated NETs (G1), well differentiated neuroendocrine carcinomas (G2) and poorly differentiated neuroendocrine carcinomas (G3) [5]. Cell proliferation activity was determined by count of Ki-67+ cells, as previously reported [34].

Patients were managed following available guidelines and recommendations [36]. Surgery was the first option of treatment in all cases, and, if residual disease was observed, adjuvant treatment with somatostatin analogs was prescribed. Patients were grouped into three categories according to their follow-up evaluation: 1) *complete remission*, if there was no evidence of tumor relapse/recurrence; 2) *stable disease*, in cases of residual but non-progressive tumor burden; and 3) *progressive disease*, if tumor growth or new lesions were detected. The Hospital's Ethics Committee approved the study, which was conducted in accordance with the Declaration of Helsinki and according to national and international guidelines. All patients signed a written informed consent before inclusion.

We obtained 72 formalin-fixed paraffin-embedded samples from primary and metastatic tissues from the 26 patients (Table 1). Of these, 30 samples corresponded to normal tissues (26 samples from adjacent non-tumoral regions and 4 normal control tissues obtained from patients that underwent intestinal, pancreatic or hepatic resection). The other 42 samples corresponded to tissues with pathological diagnosis of NET [26 from the primary site (pancreas or GIT) and 16 obtained from a metastatic site]. In order to ensure identification of representative and relevant areas of tumor and non-tumor tissues to carry out the RNA isolation of each sample, a comprehensive analysis of hematoxylin and eosin (H&E) sections was carried out by a pathologist. Simultaneously, immunohistochemical staining was carried out in paraffin-embedded blocks by the avidin-biotin peroxidase complex (ABC) method, using an anti-human chromogranin A (CgA) antiserum (Biogenex Laboratories, San Ramon, CA USA), synaptophysin, and proliferation-related Ki-67 antigen (Dako Cytomation Denmark A/S, Copenhagen, Denmark); as well as glucagon, insulin, somatostatin and gastrin. Then, tumors were classified in accordance to current guidelines [5].

### RNA isolation, reverse-transcription and quantitative real time PCR (qPCR)

Total RNA from formalin fixed paraffin-embedded (FFPE) samples was isolated using the RNeasy FFPE Kit (Qiagen, Limburg, Netherlands) according to the manufacturer's instructions. Total RNA was also isolated from cultured NET cell lines (see below) using TRIzol Reagent (Life Technologies, Barcelona, Spain) following the manufacturer's protocol and subsequently treated with DNase (Promega, Barcelona, Spain). Quantification of the recovered RNA was assessed using NanoDrop2000 spectrophotometer (Thermo Scientific, Wilmington, NC, USA). One microgram of total RNA was retro-transcribed to cDNA with the First Strand Synthesis kit using random hexamer primers (Thermo Scientific). cDNAs were amplified with the Brilliant III SYBR Green Master Mix (Stratagene, La Jolla, CA, USA) using the Stratagene Mx3000p system and specific primers for each transcript of interest. Specifically, expression levels (absolute mRNA copy number/50ng of sample) of native ghrelin, In1-ghrelin, GOAT, GHSR1a, GHSR1b, Ki67, TP53 and Bcl2 were measured using previously validated primers (Supplemental Table 1 and/or [15, 37]) and methods [15, 38]. Briefly, samples derived from human NET tissues or NET cell lines were run, in the same plate, against a standard curve to estimate mRNA copy number (1, 10^1^, 10^2^, 10^3^, 10^4^, 10^5^, and 10^6^ copies of synthetic cDNA template for each transcript) and a No-RT sample as a negative control. Thermal profile consisted of an initial step at 95°C for 3 minutes, followed by 40 cycles of denaturation (95°C for 20s) and annealing/elongation (60°C for 20s), and finally, a dissociation cycle (melting curve; 55°C to 95°C, increasing 0, 5°C/30 s) to verify that only one product was amplified. To control for variations in the amount of RNA used and the efficiency of the reverse-transcription reaction, the expression level (copy-number) of each transcript was adjusted by the expression of beta-actin (used as a control), as previously reported [39].

### Cell cultures

We used two previously validated NET cell lines: carcinoid BON-1 cells [40] and somatostatinoma derived QGP-1 cells [41]. BON-1 cell line was cultured and maintained in Dulbecco's Modified Eagle Medium (DMEM-F12; Life Technologies, Madrid, Spain) complemented with 10% fetal bovine serum (FBS; Sigma-Aldrich, Madrid, Spain) and 0.2% antibiotic (Gentamicin/Amphotericin B; Life Technologies). Meanwhile, QGP-1 was cultured and maintained in RPMI 1640 (Lonza, Basel, Switzerland), complemented with 10% FBS, 1% glutamine and 0.2% antibiotic. Both cell lines were maintained at 37°C and 5% CO_2_, under sterile conditions.

### Stable transfection of In1-ghrelin peptide

BON-1 and QGP-1 cell lines were stably transfected with pCDNA3.1 vector containing In1-ghrelin peptide (Life Technologies, Madrid, Spain) and selected as previously reported [42]. Specifically, NET cell lines were seeded in 6-well culture plates and transfected with In1-ghrelin or empty (mock) vectors using Lipofectamine 2000 Transfection Reagent (Life Technologies, Madrid, Spain) following manufacturer's instructions and selected from non-transfected cells by treatment with geneticin (Life Technologies, Madrid, Spain). Stable transfection of In1-ghrelin was always confirmed by qPCR.

### Measurements of proliferation

As previously reported [39, 42], cell proliferation of cell lines transfected with In1-ghrelin or empty (mock) vectors was measured using the alamar-blue fluorescent assay (Life Technologies, Madrid, Spain). Briefly, transfected cells were seeded in 96-well plates at a density of 3000–5000 per well and serum-starved for 12 h. Then, after 3 h of incubation with 10% alamar-blue serum-free medium, basal proliferation rate was obtained by measuring the fluorescent signal exciting at 560 nm and reading at 590 nm using the FlexStation III system (Molecular Devices, Sunnyvale, CA, USA). Subsequently, proliferation rate was similarly measured at 24, 48 and 72 h after the basal proliferation rate evaluation. Medium was replaced by fresh medium containing FBS immediately after each measurement. Results were expressed as percentage referred to control (mock transfected cells). In all experiments, cells were seeded per quadruplicate and all experiments were performed a minimum of four times.

### Measurements of migration capacity

The wound healing technique was used to assess the ability of mock and In1-ghrelin stably transfected NET cell lines to migrate, as previously reported [42]. Briefly, cell lines under confluence status and cultured in 6-well plates were serum-starved for 24 h to achieve cell synchronization, and then the wound was made using a 200 μl sterile pipette tip. Wells were rinsed using PBS and subsequently, cells were incubated for 24 h in FBS supplemented medium. Wound healing was evaluated as the area of a rectangle centered in the picture 24 h after the wound vs. the area of the rectangle just after the wound was performed. To confirm the migration assay, at least three experiments were performed in independent days, in which three pictures randomly selected were acquired along the wound per well.

### Measurements of serotonin secretion

Serotonin secretion was measured in mock and In1-ghrelin stably transfected NET cell lines using a specific commercially available ELISA kit (ALPCO, USA). Briefly, cell lines were seeded in 12-well plates at 70% confluence in serum-starved medium and 24 h later media were collected and stored at −20°C until measurements. Results are expressed as percentage of serotonin secretion of In1-ghrelin *vs.* mock-transfected cells. At least four experiments were performed in independent days, in which cells were plated per duplicate.

### Statistical analyses and Receiver Operating Characteristic (ROC) curve of the expression of the ghrelin system in NET tissues

Descriptive results were expressed as mean ± standard deviation (SD), mean ± standard error of the mean (SEM), or median ± interquartile range, as appropriate. Quantitative variables were evaluated using Spearman's bivariate correlations and differences between groups were compared using analysis of variance (U-Mann Whitney or Kruskal-Wallis ANOVA). Comparison between related variables was performed using Wilcoxon sum rank test. Samples from all groups within an experiment were processed simultaneously. *P*-values were two-sided and statistical significance was considered when *P* < 0.05. Statistical analyses were performed using SPSS 20.0 (IBM SPSS Statistics Inc., Chicago, IL, USA) and GraphPad 5.0 (GraphPad Software, La Jolla, CA, USA).

As previously reported [43], ROC was performed for evaluation of diagnostic test sensibility and specificity. Specifically, in this study ROC was used as a tool to measure how well the expression of each of the components of the ghrelin system analyzed could distinguish between different diagnostic groups [clinical outcome of patients (tumor remission, stable disease or progressive disease) and malignancy features (expression in primary tumors of patients that developed metastasis compared to those that did not develop metastasis)]. Statistical analysis of ROC curves was performed by calculating the Area under the Curve (AUC) of each transcript and comparing them with the AUC of the reference line using Student's *t*-test.

## SUPPLEMENTARY FIGURES AND TABLE


