# Supplementary material for: In1-ghrelin, a splice variant of ghrelin gene, is associated with the evolution and aggressiveness of human neuroendocrine tumors: Evidence from clinical, cellular and molecular parameters
Source: Oncotarget. 2015 Jun 18;6(23):19619–33. doi: 10.18632/oncotarget.4316 (PMC4637309; doi:10.18632/oncotarget.4316)
Supplement: Supplementary file 1 [file oncotarget-06-19619-s001.pdf]

SUPPLEMENTARY FIGURES AND TABLE

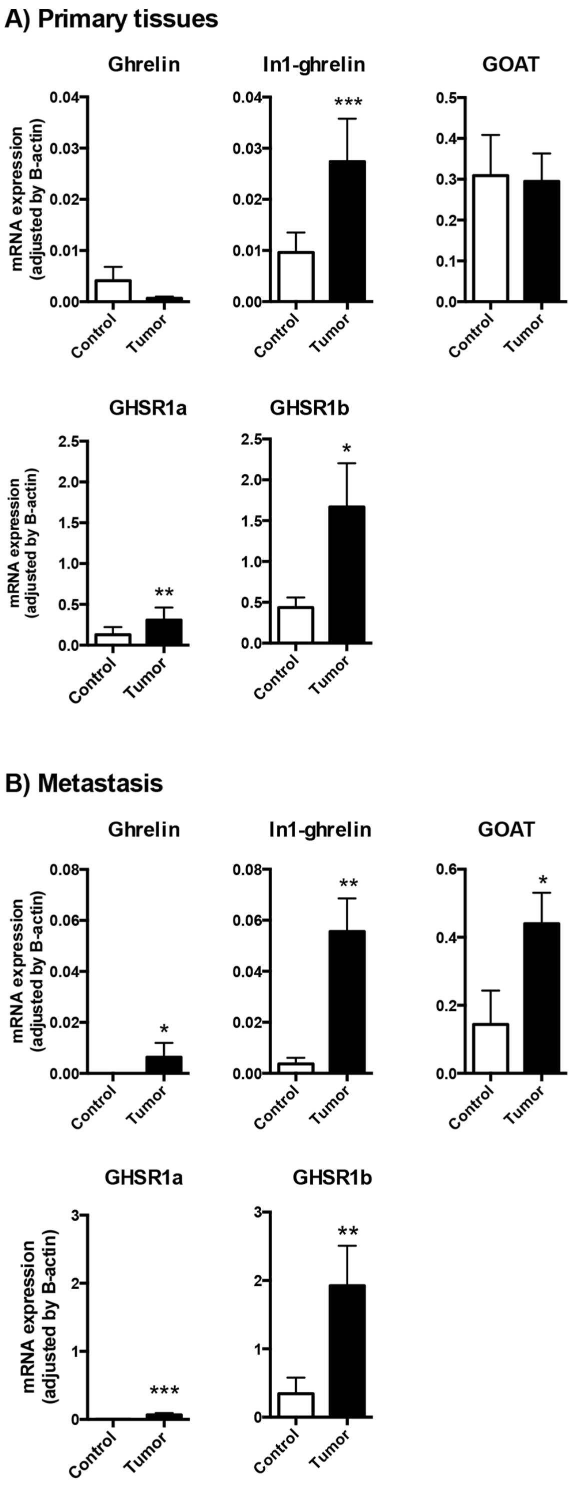

Supplementary Figure S1: Expression of ghrelin system components in NETs. A. The mRNA expression levels of the ghrelin system components (ghrelin, In1-ghrelin, GOAT, GHSR1a and GHSR1b) were determined by qPCR in primary NETs and compared to their adjacent non-tumoral control tissues. **B.** The mRNA expression levels of the ghrelin system components (ghrelin, In1-ghrelin, GOAT, GHSR1a and GHSR1b) were determined by qPCR in metastasis of NETs and compared to their adjacent non-tumoral control tissues. Data represent mean ± SEM. Asterisks (*$p < 0.05$; **$p < 0.01$; ***$p < 0.001$) indicate values that significantly differ from the adjacent non-tumoral control tissues.

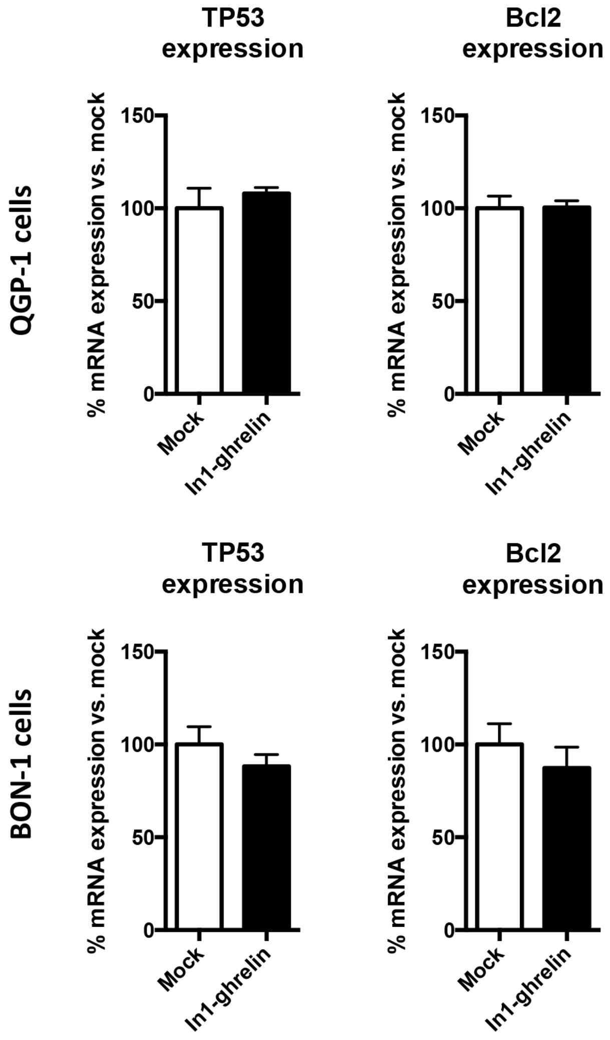

Supplementary Figure S2: Expression of apoptosis regulatory genes in NETs cell lines. The mRNA expression levels of two main apoptosis regulatory genes (TP53 and Bcl2) were determined by qPCR in In1-ghrelin transfected QGP-1 cell lines and BON-1 cell lines and compared to mock transfected control cells. Data represent mean ± SEM of $n = 3 - 6$ independent experiments.

Supplementary Table S1. Sequences and product sizes of primers used in qPCR

Amplified product	Accesion number	Sense	Antisense	Product lenght
**Native ghrelin**	NM_001134941.2	TCAGGGGTTCAGTACCAGCA	CAAGCGAAAAGCCAGATGAG	158bp
**In1-ghrelin**	GU942497.1	TCTGGGCTTCAGTCTTCTCC	GTTCATCCTCTGCCCCTTCT	215bp
**GOAT**	NM_001100916.1	TTGCTCTTTTTCCCTGCTCTC	ACTGCCACGTTTAGGCATTCT	161bp
**GHSR1a**	NM_198407.2	TGAAAATGCTGGCTGTAGTGG	AGGACAAAGGACACGAGGTTG	168bp
**GHSR1b**	NM_004122.2	GGACCAGAACCACAAGCAAA	AGAGAGAAGGGAGAAGGCACA	107bp
**B-actin**	NM_001101.3	ACTCTTCCAGCCTTCCTTCCT	CAGTGATCTCCTTCTGCATCCT	176bp
